# Elucidating the nanoscopic organization and dynamics of the nuclear pore complex

**DOI:** 10.1080/19491034.2025.2510106

**Published:** 2025-06-04

**Authors:** Kevin N. Baumann, Eva Bertosin, Anders Barth, Cees Dekker, Roderick Y.H. Lim

**Affiliations:** a Biozentrum University of Basel, Basel, Switzerland; b Department of Bionanoscience, Kavli Institute of Nanoscience, Delft University of Technology, Delft, the Netherlands; c Swiss Nanoscience Institute, University of Basel, Basel, Switzerland; d Bijvoet Centre for Biomolecular Research, Department of Chemistry, Utrecht University, Padualaan, Utrecht, Netherlands

**Keywords:** Nuclear pore complex, nucleocytoplasmic transport, super-resolution microscopy, Biomimetic approaches, computational modeling, high-speed atomic force microscopy, nanopores, transport receptors, DNA origami, selective permeability barrier

## Abstract

Due to its pivotal role as a regulator of nucleocytoplasmic transport, the structure and dynamic gating mechanism of the nuclear pore complex (NPC) is a subject of immense interest. Here, we report key recent advancements discussed at the *Selective Transport Control in Biological and Biomimetic Nanopores* meeting (Monte Verità, Switzerland, 2024) that gathered NPC experts from a range of disciplines. Novel insights were reported from cutting-edge super-resolution techniques that enable the direct interrogation of the NPC’s dynamic central transporter; computational models that unravel the mechanisms of the selective barrier; and synthetic NPC mimics as valuable *in vitro* models for delineating NPC permeability and transport dynamics. Altogether, three major insights were highlighted: (i) the presence of dynamically organised nuclear transport pathways within the NPC, (ii) the role of nuclear transport receptors that enrich and reinforce the NPC’s selective permeability barrier, and (iii) the ability of DNA origami nanostructures to mimic aspects of the NPC with unprecedented precision. Overall, the advancements marked a convergence in our understanding of NPC function by unraveling its dynamic gating mechanism at the nanoscale.

## Introduction

Nuclear Pore Complexes (NPCs) are large biomolecular machines that selectively regulate the nucleocytoplasmic transport (NCT) of diverse macromolecular cargoes between the cytoplasm and nucleus in all eukaryotic cells [1]. This process includes the import of transcription factors to and export of RNAs from the nucleus while suppressing the passage of nonspecific macromolecules – a selectivity that is striking given that this passive machine acts merely through biochemical interactions without directly consuming ATP or GTP. Defective NPC/NCT function is associated with neurodegeneration, cancer, and aging [2, 3].

Although the structural scaffold of the NPC has been resolved to near-atomic resolution [4–6], the *mechanisms* regulating the selectivity and speed of NCT within the NPC remain unclear. This has been closely linked with the behavior of phenylalanine-glycine nucleoporins (FG-Nups), whose intrinsically disordered FG-repeat domains are anchored to the scaffold surrounding the NPC’s central transporter conduit [7–10]. Cargoes are shuttled through the NPC by binding to transport factors, which in turn can interact multivalently with the FG-repeats in the NPC lumen [11–14]. Among the most extensively studied transport factors are the karyopherins (Kaps), also called importins and exportins. Emerging evidence suggests that Kaps also serve to reinforce NPC barrier function [15–19], such as by forming a ‘central plug’ that co-organizes the FG-Nups within the NPC lumen [20] – an entity whose exact composition and function has so far remained obscure. A recent discovery that is further anticipated to influence NPC barrier function is the scaffold’s ability to constrict and dilate, modulating the NPC’s inner diameter between 40 nm and 60 nm [5, 21–23]. From the pliable nanoscale structure of the NPC to the dynamic organization of the central transporter and the rapid kinetics of selective transport, these behaviors highlight the multifaceted challenges in unraveling the interplay between structural order and disorder that defines the NPC’s transport mechanism.

**Figure 1.**
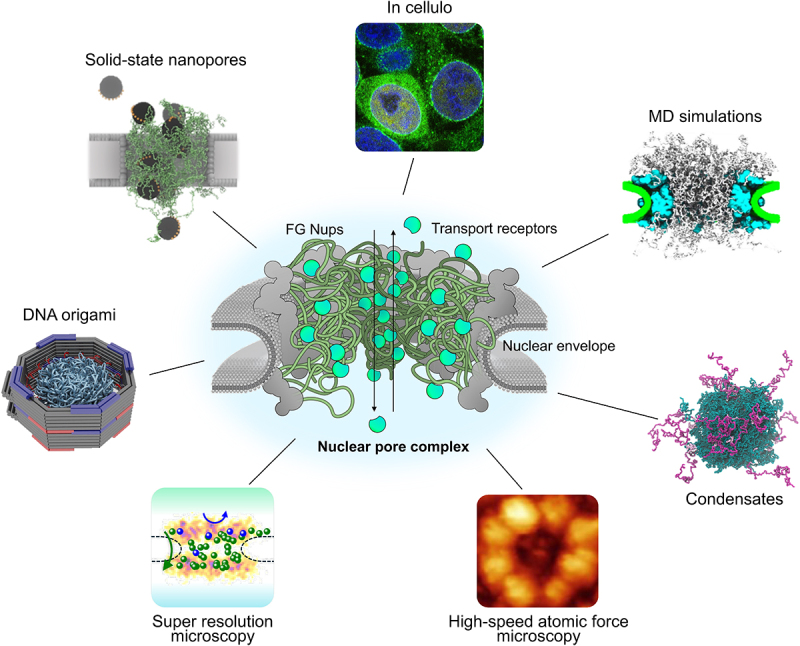
Elucidating the substructures and dynamics of the NPC. Clockwise from the top: HEK293T cells showing cytoplasmic partitioning and distinct nuclear rim staining caused by Kapβ1 (importin β1, green) enrichment in NPCs (image credit: L.E. Kapinos). MD simulations shed light on Nup-Nup interactions and transport dynamics (from [24]). Condensates can be formed by FG-Nups (from [25]). HS-AFM visualizes FG-Nup dynamics within NPCs (from [26] []). Super-resolution microscopy illuminates Kap-cargo localization within individual NPCs, revealing complete (green spheres) or aborted events (blue spheres) (image credit: S.M. Musser). DNA origami-based mimics seek to recapitulate NPC structure and function (from [27]). FG-Nup functionalized solid-state nanopores allow for high throughput analysis (from [28]). See text for details.

The *Selective Transport Control in Biological and Biomimetic Nanopores* meeting (Monte Verità, Switzerland, 2024) marked the eighth iteration of the thematic series on ‘NPC biophysics’, founded by the late Prof. Anton Zilman (see Dedication). As in previous years, it brought together an interdisciplinary group of biologists, chemists, and physicists, blending advances in theory, computation, and experimental methods. The discussions explored key mechanistic questions: What is the dynamic organization of the central transporter? How do Kaps rapidly move through the central transporter? How do large-sized cargoes such as ribonucleoprotein particles and viral capsids overcome the central transporter? What are the constituent components of the central plug? How does tethering to the NPC scaffold alter FG-Nup behavior compared to their behavior in bulk environments? What is the role of Kaps and other cofactors in maintaining the selectivity of the barrier? Where are the translocation pathways across the central transporter? How do NPC dilation and constriction influence transport? The main highlights of this meeting, describing how these questions were addressed with many complementary tools, are presented below (Figure 1).

## NPC simulations

Patrick Onck (University of Groningen) presented his lab’s work on residue-level molecular dynamics (MD) simulations of full yeast NPCs in the presence of Kap95 [29]. This work provided unprecedented theoretical insights into the dynamic organization of the central transporter and the passage of Kaps. In the absence of Kaps, the cohesive GLFG-Nups (e.g., Nup100), formed a high-density ‘ring’ along the pore scaffold. On the other hand, FxFG-Nups, such as Nsp1, formed a dynamic meshwork across the central transporter. Kap95 showed the highest affinity to the FG-rich dense ring of GLFG-Nups, where its diffusion was slowest. At higher concentrations, Kap95 increasingly localized to regions of lower FG-Nup density, i.e. the central axis of the NPC, where the mobility was higher. Intriguingly, these insights reconcile two older transport models. First, the Nsp1 pore-spanning network is reminiscent of a ‘molecular sieve’ [30], although nanosecond-long contact lifetimes suggest a much more dynamic behavior. Second, the increased size selectivity of the permeability barrier at higher Kap95 concentrations supports Kap-centric models [15, 17, 19]. Further support for Kap-centric models was presented by Rob D. Coalson (University of Pittsburgh) who studied the effect of Kap95 on the transporter NTF2 in a coarse-grained model that was also based on the Onck force field. Maurice Dekker from Onck’s lab reported on the phase separation of untethered Nups as studied by residue-level MD simulations [31]. The authors observed that GLFG-Nups, but not FxFG-Nups, formed liquid-like condensates that are stabilized by highly dynamic, short-lived FG–FG interactions mediated by cohesive FG-spacers. Despite the striking resemblance between the NPC’s GLFG-ring and the FG-Nup condensates (in terms of density and the presence of dynamic, short-lived FG–FG interactions), there was quite some discussion whether the GLFG-ring can be strictly termed a condensate – due to the different boundary conditions (tethered versus unconstrained FG-Nups).

Adapting the Onck force field, Aleksei Aksimentiev (University of Illinois at Urbana-Champaign) discussed simulations of the dynamic FG-Nup mesh tethered in yeast NPCs without Kaps [24]. To assess selectivity, they developed a method to estimate translocation rates of inert probes directly from the dynamics of the central transporter’s FG-Nup network. The energy barrier of a translocating inert probe can be approximated from the probability distribution of transient openings (‘voids’) within the disordered mesh via Boltzmann inversion. The excellent agreement of this approach with the explicit simulations of translocation trajectories highlighted the importance of the rapid dynamics displayed by the FG-Nup network. In connection to older *in vitro* data by Cees Dekker’s lab (TU Delft) on the flux of ions through FG-Nup coated nanopores, Aksimentiev provided an overview of how ionic currents can be computed from all-atom or coarse-grained simulations of biomimetic systems or even full NPCs. Notably, he also highlighted recent work comparing different all-atom force-fields for biomolecular condensates, showing that the choice of a force field can considerably affect the simulated behavior of a disordered system [32].

Barak Raveh (Hebrew University of Jerusalem) presented work on the integrative modeling of the full NPC using a newly developed framework that combines different levels of coarse-grained representations to map the design features underlying NCT [33]. To this end, they used a Bayesian approach by constraining interaction parameter values, selecting those that agreed with a large collection of diverse and verified experimental data. The resulting model reproduced well the major experimentally observed features of NCT, such as the effect of the pore milieu on FG-Nup end-to-end distances obtained from FRET, and protein density maps of the central transporter radial distributions obtained from cryo-electron tomography. While the model used a lower resolution representation of 20 amino acids per bead for the FG-Nups, it reproduced similar features to those observed by Dekker et al. [29], namely the formation of a dense ring of GLFG-Nups along the pore wall and a preferential localization of transporters in the center of the channel. The model was used to test the size dependence of passive diffusion and the effect of size and FG-binding multivalency on facilitated diffusion of transport-cargo complexes. This integrative model serves as an excellent starting point for a deeper understanding of transport through the NPC that can grow and adapt as new experimental data will become available.

## NPCs: *in vitro* to *in vivo*

David Cowburn (Albert Einstein College of Medicine) showcased the dynamic nature of FG repeat interactions [34] using an approach relying on small angle neutron scattering (SANS) and all-atom molecular dynamics (MD) simulations. In this study, the authors tracked changes to average spatial distributions between interacting domains of FG-Nups when binding to NTF2, the import factor of RanGDP. The results showed no significant inter-chain or inter-aromatic contacts that would support a static FG-Nup meshwork. In part, the spacers between the FG motifs may behave like entropic springs and hinder such static adhesive interactions. FG-Nup dynamics are likely the reason for the rapid exchange of NTF-FG motif contacts, facilitated by a ‘sliding’ of transiently bound FG motifs along the hydrophobic patches of transport receptors.

Michael Rout (Rockefeller University) provided an update on the multiscale structure of yeast NPCs at near-atomic resolution [21, 22] integrating various structural data, including cryogenic electron microscopy (cryo-EM) and crosslinking-mass spectrometry. He showed that the entire NPC is held together by flexible connectors that integrate the core scaffold, equatorial transmembrane complexes, and a lumenal ring anchoring the channel within the pore membrane. The study also uncovered an organization of the nuclear double outer ring that may be shared with ancestral NPCs. Additionally, connections between the core scaffold and the central transporter suggest localized organization at the transport machinery’s periphery, potentially coupling scaffold conformational changes to the central transporter to modulate transport.

Roderick Lim (University of Basel) reported on his lab’s use of high-speed atomic force microscopy (HS-AFM) to study FG-Nup dynamics directly within isolated yeast NPCs [20]. The work showed that the FG-Nups are in a very dynamic state and radiate from the scaffold toward Kaps in the middle of the central transporter, collectively forming an amorphous structure resembling the central plug, but also creating transient ‘voids’ in the protein density. This behavior provides structural evidence supporting the role of Kaps as functional constituents of the NPC that reinforce the permeability barrier by ‘Kap-centric control’ [13, 15, 17]. HS-AFM furthermore enabled the nanoscale examination of the morphology of *in vitro* FG-Nup hydrogels [36] to probe their selective permeability barrier-like qualities. Based on their observations of these FG hydrogels as heterogeneous aggregates of amyloid-like fibrils whose surfaces were punctuated by numerous small ‘NPC-like’ holes, they suggested that these nanoscopic holes, rather than the hydrogel body, account for its selective transport properties [20].

Siegfried Musser (Texas A&M University) presented an impressive optical visualization of Kap transport through individual NPCs at single molecule resolution. He showcased his lab’s progress to track individual cargoes and transporters in permeabilised cells with millisecond time resolution and sub-10 nm spatial resolution using super-resolution microscopy [37]. The research revealed selective transport pathways that were localized predominantly along the peripheral scaffold regions within the NPC, suggesting a blockage along the central axis – apparently in contrast to the results from the MD simulations discussed above. Using 3D MINFLUX, Musser found that individual transport trajectories followed a rapid, quasi-one-dimensional pathway, suggesting that transport was confined to a single spoke along the NPC scaffold [38]. Anders Barth from Cees Dekker’s lab presented similar MINFLUX super-resolution microscopy data, though at a less advanced stage, on isolated NPCs which showed that individual Kaps occupy distinct volumes within the central transporter and do not explore the entire NPC.

The tendency of some isolated FG-Nups to undergo liquid–liquid phase separation (LLPS) can lead to potentially cytotoxic aggregates if this process is not controlled. Liesbeth Veenhoff (University of Groningen) highlighted the roles of quality control factors associating with the FG-Nups during NPC biogenesis, thereby preventing their condensation into aggregates [25, 39]. Depleting the molecular chaperone DNAJB6 in human cells led to the accumulation of aggregated structures containing partially assembled NPCs, in so-called annulate lamellae. Depleting the FG-Nup Nsp1 in yeast cells led to NPC defects and loss of protein homeostasis. These findings suggest links between the heat shock protein chaperone network, Kaps, aging-related protein aggregation, and NPC biogenesis, which have not yet been studied [39].

Another aspect of NPC function lies in its ability to maintain the asymmetrical distribution of transport factors [19] such as RanGTP [16]. A study by Larisa Kapinos from Rod Lim’s lab asked how exportins are partitioned inside the nucleus without an NLS. This research shows that exportin 2 (CAS) N-terminus modulatesRanGTP activity to control CAS nuclear retention [40]. This points toward a separate NPC-based regulatory mechanism that is functionally independent of the NPC permeability barrier.

Pere Roca-Cusachs (University of Barcelona) presented results regarding the mechanosensitive nature of nucleocytoplasmic transport and its dependence on matrix stiffness and composition. Increases in forces applied to the nucleus enhanced NPC permeability to both passive and facilitated nucleocytoplasmic transport, with molecular weight dependence being more pronounced for passive than for facilitated diffusion [41]. Remarkably, the mechanoresponse of YAP in breast epithelial cells was found to be reduced on laminin-coated substrates [42, 43]. These results create an interesting connection between mechanosensitive nuclear transport and the invasiveness of certain cancer types [44, 47].

Another essential aspect of NPC biology that is not well understood is its biogenesis. During interphase, NPC assembly must be tightly regulated to prevent improper perforation of the NE, which could compromise the permeability barrier. To address this, Karsten Weis (ETH Zürich) described a mass spectrometry-based approach termed KARMA (Kinetic Analysis of Incorporation Rates in Macromolecular Assemblies) that was used to identify key factors involved in NPC biogenesis. This led his lab to discover that the transmembrane protein Brl1 is an essential NPC assembly factor in *S. cerevisiae* [48]. He further highlighted that Brl1’s amphipathic helical domain plays a critical role in mediating membrane fusion and ensuring correct NPC insertion into the NE. Notably, mutations in Brl1 resulted in nuclear envelope herniations and defective membrane fusion.

## Mimicking the NPC

Biomimetic approaches aim to study NPC transport by reconstructing its key aspects from the bottom up. Success in such endeavors would underscore the level of the field’s understanding of critical components, organizations, and behaviors underpinning the transport mechanism. Since NCT occurs through the central transporter, a shared feature of these approaches is that the NPC scaffold proteins are usually replaced by solid-state, synthetic materials that form a central channel in which one or more FG-Nups species can be tethered. This epitomizes the close synergies between NPC function, smart polymers, and synthetic nanopores. While these NPC mimics are developed as toy models for understanding NPC transport, they may also be used in emergent technologies such as DNA sequencing, water purification, and biological sensing [45].

Cees Dekker (TU Delft) reported the use of zero-mode waveguides (ZMW) to investigate transport through FG-Nups. Briefly, ZMWs are nanopores in a solid-state material that can either be coated with a metal layer (such as gold-coated polycarbonate supports) or made entirely of metal, using materials like gold or palladium [46]. These metal nanopores block the propagation of light with wavelengths larger than the pore diameter, resulting in an evanescent wave that penetrates only a small portion of the nanopore. As a result, the bulk solution above the metal support is not illuminated, while fluorophores can be excited and detected as they cross the nanopore. Specifically, nanopores with diameters comparable to the NPC size were functionalized with FG-Nups, and the diffusive transport of fluorescently labeled proteins was examined [49] – showing a clear diameter dependence of the transport with different behavior for pores larger or smaller than 55 nm.

Fabien Montel’s group (Ecole Normale Supérieure de Lyon) also used ZMWs to study the directionality [50] and selectivity [51, 52] of transport through nanopores. One advantage of this approach was the multiplexing capabilities, as up to 10^4^ pores could be visualized in a single field of view [53]. Upon applying pressure to induce transport, they investigated the effect of various grafted polymers, including hydrophilic and hydrophobic polymers of different lengths, and tested their behavior depending on temperature [52] as well as interactions between these polymers and proteins, DNA [50,53], RNA, viruses [54,55]^56^, and artificial polymers [57].[14]

DNA origami is a powerful technique to fold a long single-stranded DNA molecule into virtually any desired 2D or 3D shape [58], and it has been applied as a scaffold for biomimetic NPCs. DNA origami structures can be relatively easily functionalized with proteins, fluorophores, or hydrophobic moieties to facilitate the integration into lipid bilayers. Due to its versatility, both the labs of Chenxiang Lin (Yale School of Medicine) and Cees Dekker employed the origami technique to anchor FG-Nup proteins in an NPC-like pore geometry, controlling the number, types, and positions of FG-Nups attached to the inner lumen [56, 59, 60]. Macromolecular complexes as large as HIV viruses can interact with the FG-Nup barrier anchored into these artificial NPCs [61]. The upcoming challenges in this field involve developing structures that can mimic the recently discovered NPC expansion and dilation [5] to analyze its impact on transport. This can be accomplished in two ways: either by constructing a set of structures with different fixed diameters [27] or by building compliant structures that can expand and contract [62, 63, 64]. On this note, Chenxiang Lin described the design of expandable DNA origami nanopores that could be used as force sensors to test for the interactions between FG-Nups. Eva Bertosin from Cees Dekker’s group presented similar work on DNA origami nanopores that can be expanded and contracted using the addition of various DNA oligonucleotides. Another current challenge involves integrating DNA origami nanostructures into lipid membranes. One solution is to conjugate the structure with cholesterol moieties. Alternatively, Lin’s group leverages a bacterial toxin, i.e. pneumolysin, to form large nanopores as an adapter for the DNA origami pores.

Towards building fully artificial NPC-like systems, Andreas Dahlin (Chalmers University of Technology) anchored pH-responsive and thermo-responsive polymeric brushes to surfaces to determine general transport principles. This approach may enable to study, for instance, whether cohesiveness is a necessary or sufficient requirement for selectivity. These systems have applications beyond the study of the NPC. For instance, nanochambers coated with polymer brushes can be used to trap macromolecules reversibly for optically analyzing interactions involving multiple proteins [64]. Furthermore, the polymer brushes exhibit antifouling properties against protein binding [65].

## Summary and outlook

The meeting served as an important forum to discuss the state of the field. In this final section, we summarize new developments, key research questions, and future directives. Certainly, the ability of the NPC to dilate and constrict is of urgent research interest. While the diameter of the central channel has been observed to expand in response to external stress [5] and during the transit of exceptionally large cargoes [66], isolated NPCs adopt a more contracted state within the typical range of NPC diameters [21]. The processes that modulate the NPC diameter and how they are regulated remain unknown. It is also still largely unknown whether individual cells contain both dilated and contracted NPCs, potentially reflecting different functional states. Due to the anchoring of the NPC scaffold to the nuclear envelope, membrane tension has been speculated to play a role in NPC dilation [5]. Andreu et al. reported direct links between the cytoskeleton and several structured components of the NPC to form the connection between external stress and forces [41]. *In vitro* studies presented by Lin revealed another element by showing that the FG-Nup concentrate itself can exert contractile forces on a suspended, expandable DNA origami ring. Also, the transport selectivity of Kap95 shows to be influenced by the pore diameter in NPC mimics [49]. Hence, it will be important to understand how NPC diameters are regulated to balance selective transport and barrier function according to the functional needs of the cell.

Standing the test of new experimental data, coarse-grained models will significantly increase in their predictive potential. Consequently, ever more mature computational models have the potential to go beyond descriptive, coarse-grained representations of the NPC toward more accurate and, importantly, predictive modes to provide novel insights and guide future experiments. Overall, the recent advancements depart from earlier phenomenological models that borrow from bulk concepts derived from macroscopic assemblies to describe the behavior of disordered FG-Nups within the central transporter. Such macroscopic assemblies are increasingly disfavored as relevant model systems because of obvious mismatches in size, composition, and organization with respect to the NPC. For example, FG hydrogels [36, 67,68], which are used as model systems to describe a cross-linked meshwork or ‘selective phase’, show static interlaced amyloid structures [69] that conflict with the highly dynamic nature of the central transporter [20, 35, 70]. On the other end of the spectrum, the central transporter is often referred to as a ‘biomolecular condensate’ [71, 72–75]. However, within the NPC, the FG-Nups are anchored in a defined stoichiometry, creating a scenario that differs significantly from the *in vitro* LLPS of isolated FG-Nups in solution, where molecules dynamically exchange between dense and dilute phases [31]. Beyond nomenclature, the question remains how insights from bulk condensates or hydrogels can provide insights into the structure and functioning of the exquisite nanoscopic organization and composition of the central transporter.

The situation is further complicated if additional components such as Kap receptors and cargoes in transit are added to the mix, yielding a complex NPC structure for which Anton Zilman had previously put forward the term ‘dumetum’ (Latin for ‘thicket’). The disordered and chaotic nature of this thicket serves as a reminder of our still limited understanding of the mechanistic details of nuclear transport. Nevertheless, one clear takeaway from this meeting is the growing consensus among attendees regarding many features of the NPC transport mechanism. Much of the somewhat stalled debate about the first generation of early models was left behind, and the convergence of ideas suggests that future research efforts may result in the community reaching a consensus that accurately reflects the intricacies of nucleocytoplasmic transport. Cutting-edge techniques that enable real-time and super-resolved observations of the NPC in all its dynamics will be of particular relevance and are already gaining momentum in the field.

## Data sharing statement

Data sharing is not applicable to this article as no new data were created or analyzed in this study.

## Dedication

We pay tribute to our friend and colleague, the late Prof. Anton Zilman, who founded this series of ‘NPC biophysics’ meetings, and whose loss in 2024 was both a personal and scientific blow to our field. We owe him a tremendous debt of gratitude for bringing our community together and fostering collaboration. Though Anton is no longer with us, his vision – the pursuit of ‘simplicity’ in understanding NPC function – endures through our collective work and in our remembrance of him. His thoughts, laughter, and camaraderie will be sadly missed.
